# Traditions and plant use during pregnancy, childbirth and postpartum recovery by the Kry ethnic group in Lao PDR

**DOI:** 10.1186/1746-4269-7-14

**Published:** 2011-05-10

**Authors:** Vichith Lamxay, Hugo J de Boer, Lars Björk

**Affiliations:** 1 Department of Systematic Biology, Evolutionary Biology Centre, Uppsala University, Norbyvagen 18D, SE-75236 Uppsala, Sweden; 2 Department of Biology, Faculty of Sciences, National University of Laos, Dongdok campus, Vientiane, Lao PDR

## Abstract

**Background:**

Activities and diet during the postpartum period are culturally dictated in many Southeast Asian cultures, and a period of confinement is observed. Plants play an important role in recovery during the postpartum period in diet and traditional medicine. Little is known of the Kry, a small ethnic group whose language was recently described, concerning its traditions and use of plants during pregnancy, parturition, postpartum recovery and infant healthcare. This research aims to study those traditions and identify medicinal plant use.

**Methods:**

Data were collected in the 3 different Kry villages in Khammouane province, Lao PDR, through group and individual interviews with women by female interviewers.

**Results:**

A total of 49 different plant species are used in women's healthcare. Plant use is culturally different from the neighboring Brou and Saek ethnic groups. Menstruation, delivery and postpartum recovery take place in separate, purpose-built, huts and a complex system of spatial restrictions is observed.

**Conclusions:**

Traditions surrounding childbirth are diverse and have been strictly observed, but are undergoing a shift towards those from neighboring ethnic groups, the Brou and Saek. Medicinal plant use to facilitate childbirth, alleviate menstruation problems, assist recovery after miscarriage, mitigate postpartum haemorrhage, aid postpartum recovery, and for use in infant care, is more common than previously reported (49 species instead of 14). The wealth of novel insights into plant use and preparation will help to understand culturally important practices such as traditional delivery, spatial taboos, confinement and dietary restrictions, and their potential in modern healthcare.

## Background

Medicinal plants have a significant role during pregnancy, birth and postpartum care in many rural areas of the world. Plants used in women's health related conditions such as female fertility, menorrhea, birth control, pregnancy, birth (parturition), postpartum (puerperium) and lactation, including infant care, have been documented for various ethnic groups (e.g. [1-6]). Research focusing on the use of these plants often focuses on the realm of knowledge of male traditional healers, and scholars have missed the wealth of knowledge that is held by women [7].

Pregnancy, parturition and the puerperium each mark a significant step in *matrescence *[8], and are not without risk to the mother and infant. According to the latest data for Lao PDR, the infant mortality rate (deaths per 1000 live births) and maternal mortality (maternal deaths per 100 000 live births) is respectively 60.3 and 660 [9,10], with mortalities likely to be higher in remote areas. By comparison, those numbers for Sweden are 3.2 and 3.0 [9,10].

These cultural traditions, such as postpartum confinement, steam baths and food taboos, are common and widespread in Southeast Asia, and form the core of primary maternity healthcare in many rural areas in Laos. In the context of the introduction and modernization of primary healthcare systems in rural areas, and with training programs for traditional birth attendants focusing on the paradigms of Western medicine, this traditional knowledge has often been ignored [11]. Previous studies have even expressed concern over possible negative effects of traditional postpartum practices, such as discarding the colostrum, food taboos leading to undernourishment of the mother, and early weaning due to a perceived lack of breast-milk [12,13]. Erosion and deterioration of traditional medical knowledge can be observed in many cultures and leads not only to a loss in biocultural diversity, but also diversity in alternatives for primary healthcare [14]. Documenting the use of plants and elements of traditional birth practices by ethnic minorities is not only an important aspect of understanding and analyzing these practices, but a way to perpetuate knowledge at risk of being lost.

Previous work by our group [15] focused on all plant use during pregnancy, parturition, and postpartum for lactation and postpartum recovery among three poorly studied ethnic groups, the Brou, Saek and Kry, in Khammouane province, Lao People's Democratic Republic. All three groups are ethno-linguistically more closely related to groups living in other areas. The Kry are hypothesized to be the earliest of the current inhabitants. The Kry were followed by the Saek arriving some 300 years ago from just across the current Vietnamese border, followed by the Brou arriving over the course of the last century or so from lower areas along the Korat Plateau in present-day Thailand [16]. Saek speakers came in search of flat irrigable land on which to grow wet rice crops [17]. Traditions and plant use surrounding childbirth for these three ethnic groups are poorly understood [15], and what little is known is mainly anecdotal [18-20].

The Kry are a group of about 300 people living in the upper reaches of the Nam Noi valley, in the Nakai-Nam Theun National Biodiversity Conservation Area, Khammouane Province, Laos. They live within a day's walk of the Vietnamese border at Ha Tinh Province. The villages lie between 600 m and 700 m above sea level, just on the Western side of the Annamite mountain range. The Nam Noi valley lies in the path of shortest distance anywhere in Laos from the Mekong to the South China Sea, and for this reason, the area has long been a trade route, as documented in Vietnamese administrative archives since the early 17th century [21]. The Kry language belongs to the Vietic sub-branch of Eastern Mon-Khmer in the Austroasiatic language family, and was recently described [17].

The Kry are animists and have traditionally lived a nomadic live-style in small bands as hunter-gatherers. Settlement in and near villages of Lao and other ethnic groups in recent decades has led to a shift in traditions, and currently the Kry live in houses made of bamboo raised on poles and practice subsistence shifting-cultivation of rice and vegetables, as well as some minor irrigated paddy rice, similar to other groups living in the area. This process had led to cultural amalgamation, and today few communities exist with a majority Kry population [18].

Kry everyday life includes ritual taboos that people are subject too, most notably the forbidding of certain people, at certain times, from going up into certain houses at all. For instance, when a woman is menstruating, she is not to ascend any house but must 'stay down below' or 'stay down on the ground'. At these times she sleeps in a separate menstruation hut. Other forms of contamination can keep people down on the ground too. For instance, a husband assisting during childbirth is not allowed to ascend any house in the village other than his own house until such time as his contamination is resolved by formal ritual [20].

The data presented in this study builds on our previous study [15], but focuses specifically on the Kry, the least studied ethnic group in the Nakai-Nam Theun area. The main research questions posed are 1) what are the Kry childbirth and postpartum practices and rituals, and 2) how do these practices and rituals differ from the Brou and Saek ethnic groups. Following research questions 1 and 2, we tested the hypothesis that cultural consensus based on postpartum plant use is affected by underlying variation in cultural traditions. The study provides a detailed overview of medicinal plant species used in women's healthcare; and describes the unique cultural traditions surrounding pregnancy, childbirth and postpartum recovery observed by this group of people. The data may aid in the development and implementation of culturally sensitive and appropriate healthcare by the Lao government or non-governmental organizations working in this field.

## Materials and methods

### Study site

**Figure 1:**
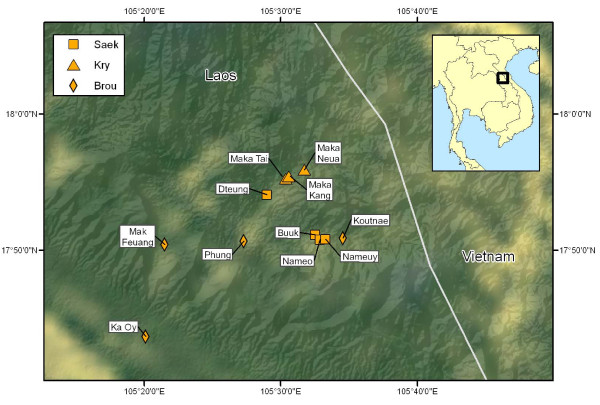
**Map of the study area**. Including all villages from this study and de Boer & Lamxay [15]. Icons represent: Brou (Diamond), Saek (Square), and Kry (Triangle). Note: The Brou village Ban Ka Oy no longer exists in its current location. Map created by Anders Larsson.

The data presented here, are independent from [15], and were collected during three expeditions in June 2008; July 2009, and July 2010 in 3 Kry villages in the Annamite Mountains in the Nakai-Nam Theun National Biodiversity Conservation Area, Nakai District, Khammouane Province, Lao People's Democratic Republic: Maka Tai (N 17 ° 56' 11.8", E 105 ° 31' 45.2", Altitude 634 m., Population 38, Number of households 11), Maka Kang (N 17° 55' 59.8", E 105° 33' 14.8", Alt. 642 m., Pop. 120, N° households 22), and Maka Neua (N 17° 55' 25.9", E105° 30' 35.2", Alt. 613 m, Pop. 143, N° of households 25); all located along the Nam Maka in the Nam Noi valley; above the Nakai Plateau (Figure 1). Note that various transliterations and versions exist for the name of the Kry ethnic group (some of which encompass more groups than solely the Kry): *Kry*, Kree, Kri, Salang, Makaa, Labree, Yubree, Arehm.

### Interviews

Data collection was done using the following general format: interviews were conducted in the homestead. After introducing the research team and research objectives to the head of the village, an informal open-ended interview was conducted to collect demographic and social data about the village, followed by a mixed gender group interview led by the first author as a means of brainstorming on the subject. The following day, two group interviews were conducted with people selected by the head of the village as knowledgeable on plant use or childbirth customs: one with male informants by male interviewers; and another with female informants by female interviewers: the village midwife, nurse and knowledgeable women with one or more child. The interviews focused on pregnancy, childbirth and labor, and in addition plants used in women's healthcare and for treating diseases in children. Group interviews were culturally readily acceptable, but valuable data may have been overlooked, as verbal dominance may not correlate with traditional knowledge. Group interviews were followed up by individual interviews with women at their homesteads by the female interviewers to triangulate data from the women group interviews, and elicit additional data on childbirth traditions and rituals. Upon completion of the interviews gender-separated group walks were made in the surrounding forest to collect the plants mentioned during the interviews. Some additional information was recorded while pressing the plants for herbarium vouchers. A total of 20 informants, 13 female and 7 male, were interviewed during group interviews, and 10 individual interviews were carried out, representing about 30% of all Kry households. All interviews were conducted in Lao.

### Botanical collections

Plant names mentioned during the interviews were recorded in Lao and transliterated from Kry to Lao script or Roman script using French phonetics as is common in Laos. Plant material was collected, pressed and drenched in alcohol for herbarium vouchers and subsequent identification. A complete set of herbarium vouchers was deposited at the herbarium of the Department of Biology of the National University of Laos and at the Uppsala University Herbarium (UPS). Common cultivated species were identified in the field, using the local name, and/or a checklist of Lao and scientific names (Callaghan, 2004). Species and author names follow the Checklist of the Vascular Plants of Lao PDR [22].

### Data analysis

Anthropac 4.98 [23] was used to re-analyze the data from [15]. All reported species used in postpartum healthcare for the Kry were combined with the data from [15], loaded, dichotomized, tested for similarity using positive matches [23,24]. Data were plotted using non-metric multidimensional scaling, in which Euclidian distances between all the points in the similarity matrix are computed and the data are represented in a 2-dimensional space in an optimal way [24,25].

## Results

### Kry ethnic variation

Respondent data established a variation in childbirth traditions among the Kry, as informants often responded that the *Kry Thae *(Genuine Kry) followed a practice in a certain way, whereas the *Kry Phong *(Fallen Kry) observed it differently. Further inquiry revealed that the Kry Phong are defined as people that are either: a) outsiders that have married into the Kry, but practice some of their own traditions; or b) Kry people that have moved to the villages from elsewhere, mainly Vietnam, and practice some of their own traditions. The main distinguishing characteristic of a Kry Phong household is the positioning of the menstruation hut as an annex to the main house, and the postpartum practice of *mother roasting *(a treatment in which the mother lies for 30 - 60 minutes on a bed over a hot charcoal brazier [15]). The medicinal plant species used were the same for both groups, with the Kry Phong using species in decoctions for *hotbed *(the practice in which the mother rests during recovery on a bed continuously warmed over charcoal brazier), steamsauna and bathing, and the Kry Thae using the same species in decoctions for consumption. Roughly 20% of the Kry households in the three Maka villages had menstruation huts as an annex to the main house using a separate ladder. Traditions of the Kry Thae are presented below as Kry traditions, and practices of the Kry Phong are discussed at the end of each section if these differ from the previous.

### Plant use

**Figure 2:**
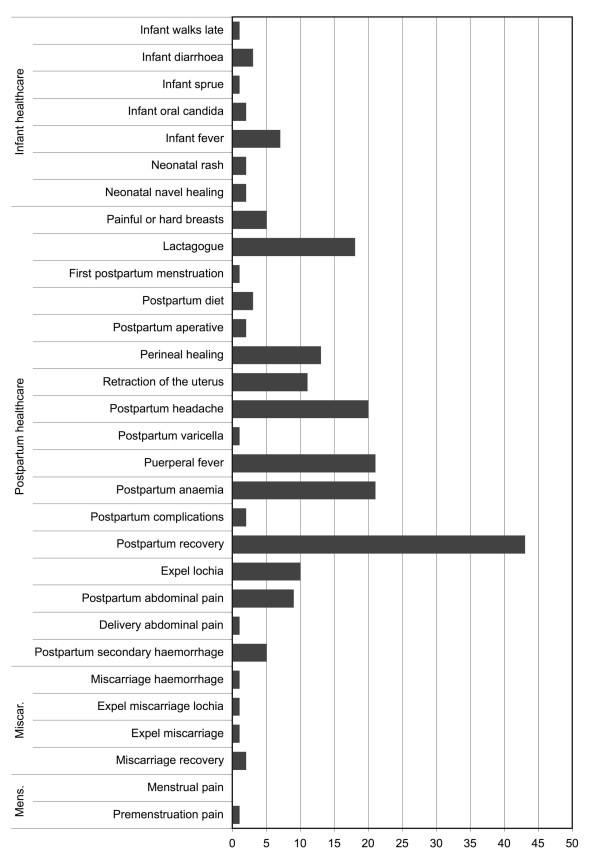
**Importance of medicinal plant use in postpartum healthcare**. Number of plants mentioned per ailments

Medicinal plant use in women's health is common and widespread among the Kry. The use of medicinal plants is generally avoided during pregnancy, but once the infant is born, both mother and infant use a variety of medicinal plants. During interviews the preparation and use of 49 plant species around childbirth were reported (Table 1). Many species are combined with others in mixtures where they constitute essential ingredients, sometimes substituted for others with similar medicinal properties (Table 2). The uses can be broadly classified into menstruation cycle, parturition (delivery), postpartum recovery, breast-feeding, and neonatal healthcare and plant use per class can be subdivided into different conditions (Figure 2).

**Table 1:** Kry medicinal plants used in women's healtcare

Scientific name	Lao name	Kry name	Vouchers	Part used	Preparation	Medicinal use
*Ageratum conyzoides *L. Asteraceae	ຫຍ້າຂິວ Nga Kiou	ເປິລໂຮຍ; ເປິລຂິວ Peuale Hoey; Peuale Kiou	VL 1445; VL 1571; VL 1758; VL 1842; Kool 501; Kool 518; Kool 634; Kool 653	Roots	Decoction-drink	Postpartum recovery 1st phase: Perineal healing; Retraction of the uterus

*Alpinia galanga *(L.) Willd. Zingiberaceae	ຂ່າ Kha	ໂປຣບ; ໄປຣ Prorbe, Prai	VL1748; VL 1383; VL 1776; VL 1854	Rhizome; Leaves; Pseudostems	Roast-Eat; Decoction-drink	Postpartum recovery 1st phase; Postpartum diet; Postpartum recovery 1st and 2nd phase: Anaemia (dizziness, headache); Mild puerperal fever; Lactagogue

*Amaranthus spinosa *L. Amarantaceae	ຫົມໜາມ Hom Nam	ກວາດ ປຣີ Kouad Pri	VL 2148	Roots	Cold-infusion-drink	Postpartum varicella

*Amomum microcarpum *C.F. Liang & D. Fang Zingiberaceae	ໝາກແໜ່ ງຄຳ Makneng Kham	ໄປຣໝາກແໜ່ ງດີດີ Pray Makneng Didi	VL 1371; VL 1763; VL 1828; VL 2039; Kool 503; ELLA 13; ELLA 16	Leaves; Pseudostems	Decoction-wash	Infant fever, reduces temperature

*Artocarpus heterophyllus *Lam. Moraceae	ໝາກມີ້ Mak Mi	ໝາກມີ້ Mak Mi	VL 2125; Kool 472; ELLA 50	Powdered Bark; Leaves	Cold apply; Decoction-drink	Neonatal navel healing; Lactagogue

*Barringtonia longipes *Gagnep. Lecythidaceae	ມົມຍານ Nomngan	ມົມຍານ Nomngan	VL 1485; VL 1757	Leaves	Roast-warm poultice or massage	Lactagogue; Painful or hard breasts; Improve flow of milk

*Bischofia javanica *Blume Euphorbiaceae	ສົ້ມຝາດ Somphat	ຈາລັງເດິມ; ເກຼິນສົມຝາດ Jalangdeum; Kreuale Somphat	VL 1752; VL 2043	Leaves	Roast-poultice or massage; Crush-cover or poultice	Lactagogue; Painful or hard breasts; Improve flow of milk; Neonatal navel healing

*Blumea balsamifera *(L.) DC. Asteraceae	ນາດ Nad	ຕຼິງ Tring	VL1751; VL 1824; Kool 614; ELLA 49; ELLA 57; ELLA 78	Leaves; Stems	Decoction-drink	Postpartum recovery 1st and 2nd phase; Perineal healing; Retraction of the uterus; Miscarriage recovery

*Butea monosperma *(Lam.) Taub. Fabaceae	ເຄຶອໄຊຊ້າງ Kheua Sai Xang	ກສິໄຊຊາງ Ksi Sai Xang	VL 2133	Leaves	Cold infusion-drink	Infant diarrhoea

*Calamus rudentum *Lour. Arecaceae	ບຸ່ນ Boun	ກສິປີເອິລ Ksi Pieule	VL 1756; Kool 668	Shoots	Roast-eat	Postartum recovery 1st phase; Postpartum diet; Lactagogue

*Callicarpa arborea *Roxb. Verbenaceae	ກະພາ Kapha	ກະພາ Kapha	VL 2036	Sapwood; Innerbark	Roast-decoction-drink	Postpartum recovery 1st and 2nd phase; Expel lochia; Postpartum abdominal pain; Perineal healing; Retraction of the uterus

*Castanopsis indica *(Lindl.) A.DC. Fagaceae	ກໍ່ໜາມ Ko Nam	ເກຼິນກໍ່ Kreuale Ko	VL 1468	Leaves	Roast-decoction-drink	Postpartum recovery 1st and 2nd phase: Postpartum secondary haemorrhage; Perineal healing; Retraction of the uterus

*Catunaregam spathulifolia *Tirv. Rubiaceae	ໝາກສັກ; ໝາກໜາມຄັກ Maksak; Mak NamKhak	ໝາກຄັກ Makkhak	VL 1340; VL 1463; VL 1514; VL 1552	Stems; Sapwood	Roast-decoction-drink; Cold-infusion-drink	Postpartum recovery 1st and 2nd phase: Expel lochia; Perineal healing; Retraction of the uterus; Abdominal pain; Varicella

*Centella asiatica *(L.) Urb. Apiaceae	ຜ້ກໜອກ Pak Nok	ກວາດ ຕຈອກນອກ Kouad Tchoknok	VL 1817; VL 1393; Kool 523; Kool 553	Whole plant	Crush-poultice	Infant high fever, reduces temperature

*Choerospondias axillaris *(Roxb.) B.L.Burtt & A.W.Hill Anacardiaceae	ໝາກມຶ Mak Meua	ໝາກມຶ Mak Meua	VL 2039; VL 1770a	Inner bark; Stems	Roast-decoction-drink	Postpartum recovery 1st phase: Expel lochia; Perineal healing; Retraction of the uterus; Postpartum secondary haemorrhage; Postpartum recovery 2nd phase: Anaemia (dizziness, headache); Mild puerperal fever

*Chromolaena odorata *(L.) R. King & H.Robinson Asteraceae	ຫຍ້າຝຣັ່ງ Nga Frang	ເປຼິລ ເຍີຣະມັນ Peuale Ngeuraman	VL 1787; Kool 540; Kool 632; Kool 648	Roots	Decoction-drink	Premenstruation pain; Postpartum recovery 1st and 2nd phase: Perineal healing; Retraction of the uterus; Expel lochia

*Cissus repens *Lam. Vitaceae	ເອັນອ່ອນ En On	ກສິ ເອັນອ່ອນ Ksi En On	VL 1843; VL 1346; VL 1484; VL 1564	Twigs; Leaves	Roast-warm poultice or massage	Infant is late to learn walking

*Diospyros apiculata *Hiern Ebenaceae	ເຂຶອເຖຶ່ອນ Kheuateuan	ເຂຶອເຖຶ່ອນ Kheuateuan	VL 1465; VL 1536	Ripe fruits	Fresh-eat	Abortifacient

*Dracaena angustifolia *(Medik.) Roxb. Agavaceae	ຄອນແຄນ Khonkhen	ປ້ອງ ຄອນແຄນ Pong Khonkhen	VL 1449; VL 1535	Leaves	Boil-steamsauna	Postpartum recovery 2nd phase: Restorative/aperative; Puerperal fever; Physical recovery

*Embelia ribes *Burn. Myrsinaceae	ເຄຶອເລຶອດ Kheua Leuat	ກສິ ເລຶອດ Ksi Leuad	VL 1739; VL 2035; Kool 500	Stems; Roots	Decoction-drink; Roast-decoction-drink	Postpartum recovery 1st phase: Abdominal pain, Expel lochia; Postpartum recovery 2nd phase: Postpartum bleeding; Perineal healing; Retraction of the uterus; Physical recovery

*Ficus hispida *L.f. Moraceae	ເດຶ່ອປ່ອງ Deuapong	ເກຼິລ ເດຶ່ອປ່ອງ Keuale Deuapong	VL 1810; VL 1548; VL 1821; Kool 511	Stems	Cold-infusion-drink; cold-infusion-wash	Neonatal rash after high fever

*Glochidion eriocarpum *Champ. Euphorbiaceae	ກຳບໍ່ສຸກ Kambosouk	ເກຼິລ ກຳບໍ່ສຸກ Kreuale Kambosouk	VL 1779; VL 1404; Kool 469	Roots; Twigs; Leaves	Decoction-drink	Postpartum recovery 1st and 2nd phases: Anaemia (dizziness, headache); Mild puerperal fever; Abdominal pain

*Gonocaryum lobbianum *(Miers) Kurz Icacinaceae	ກ້ານເຫລຶອງ Kanleuang	ແສນເມຶອງ Sengmouang	VL 1396; VL 1424; VL 1516; Kool 470	Twigs; Leaves	Roast-warm poutice or massage	Lactagogue; Painful or hard breasts; Improve flow of milk

*Hedychium *sp. Zingiberaceae	ຊາຍເຫິນ Sayheuan	ລາງຍາງ Langyang	VL 1745; VL 1829; VL 2031; Kool 506	Rhizome; Shoots	Decoction-drink; Fresh crush-drop in mouth of infant	Postpartum recovery 1st and 2nd phases: Anaemia (dizziness, headache); Mild puerperal fever; Lactagogue; Infant oral candida; Infant fever

*Houttuynia cordata *Thund. Saururaceae	ຄາວທອງ Khaothong	ກວາດ ຄາວທອງ Kouad Khaothong	VL 1381	Whole plant	Crush-poultice	Infant fever, reduces temperature

*Lagerstroemia calyculata *Kurz Lythraceae	ລານ; ເປຶອຍ Lan, Peuay	ກາຣອງປຣູ Ka Rong Prou	VL 1809; Kool 547; Kool 679	Innerbark	Roast-decoction-drink	Postpartum recovery 1st and 2nd phases: Expel lochia; Perineal healing; Retraction of the uterus

*Macaranga denticulata *(Blume) Müll.Arg. Euphorbiaceae	ບໍຫູຊ້າງ Po Houxang	ຕາ ວຢົໂທດ Ta Yuathoh	VL 1754; VL 1553	Stems; Wood	Decoction-drink	Postpartum recovery 2nd phase: Postpartum emmenagogue; Postpartum first menstruation (3rd phase)

*Maesa *spp. Myrsinaceae	ເດີນ Deuan	ເດີນ Deuan	VL 1861; VL 1869; VL 2041 Kool 543; Kool 842	Leaves; Stems	Roast-cold infusion-drink; Roast-decoction-drink	Infant diarrhoea; Infant fever

*Mallotus barbatus *Müll.Arg. Euphorbiaceae	ບໍຫູ Po Hou	ຕະຈຶຣັງ ຕົວ ຢົວ Tchirang Tua Yua	VL 2049	Roots	Roast-decoction-drink	Postpartum recovery 1st and 2nd phase: Postpartum abdominal pain, Postpartum secondary haemorrhage; Infant sprue

*Musa acuminata *Colla Musaceae	ໝາກປີ; ປີກ້ວຍ Mak Pi, Pi Kouay	ໝາກປີ Makpi; Ta Lou Ma La	VL 1755	Inflorescences; Young pseudostems	Roast-eat; Soup-eat	Pospartum recovery 1st phase: Postpartum diet; Lactagogue; Pregancy diet for easy delivery

*Neonauclea purpurea *(Roxb.) Merr. Rubiaceae	ສະໂກ Saco	ທາໂກ Tako	VL 1750; VL 1782; VL 1832	Leaves; Bark	Roast-decoction-drink	Infant fever, reduces temperature; Infant diarrhoea

*Phoebe lanceolata *(Nees) Nees Lauraceae	ພາຍເວັ້ນ Phayven	ເກຼິລ ພາຍເັ້ນ Kreul Phaiven	VL 1353; VL 1511; Kool 538; Kool 619	Leaves	Warm poutice; Massage	Lactagogue: Painful or hard breasts; Improve flow of milk

*Polyalthia cerasoides *Benth. & Hook. Annonaceae	ນ້ຳເຕົ້ານ້ອຍ Namtaonoi	ນ້ຳເຕົ້ານ້ອຍ Namtaonoi	VL 1504; Kool 513; Kool 575	Stems	Decoction-drink	Postpartum recovery 1st and 2nd phase: Anaemia (dizziness, headache); Mild puerperal fever

*Psidium guajava *L. Myrtaceae	ໝາກສີດາ Mak Sida	ສີດາ Sida	VL 1344	Shoots; Leaves	Cold infusion-drink	Infant diarrhoea

*Psychotria sarmentosa *Blume Rubiaceae	ຫວ້ານຈອດ Vanchod	ເກຼິລ ວານຈອດ Kreuale Vanchod	VL 1762; VL 1332; VL 1500; VL 1737; VL 1820; VL 1860; VL 2045; Kool 468	Roots; Stems	Roast-decoction-drink	Postpartum recovery 1st and 2nd phase: Physical recovery; Lactagogue; Perineal healing; Retraction of the uterus

*Rhapis laosensis *Becc. Arecaceae	ສານ Sane	ກອລ ຈູລ Koile Jule	VL 1439; VL 1481; Kool 571	Roots; Stems	Roast-decoction-drink	Postpartum recovery 1st phase: Anaemia (dizziness, headache), Puerperal fever, Blood loss, Weakness; Expel lochia; Miscarriage recovery: Miscarriage bleeding

*Rubus cochinchinensis *Tratt. Rosaceae	ກະທຸ້ມແດງ Katoum Deng	ກສິ ກະທູມນ້ອຍ Ksi Katoun Noi	VL 1744; VL 1373; VL 1494; VL 1803; Kool 574	Roots	Roast-decoction-drink	Postpartum recovery 1st and 2nd phase: Anaemia (dizziness, headache); Mild puerperal fever

*Rubus tonkinensis *F.Bolle Rosaceae	ກະທຸ້ມຂາວ Katoum Khao	ກະທຸ້ມໃຫຍ່ Katoum Nhai	Kool 552; Kool 631	Roots	Roast-decoction-drink	Postpartum recovery 1st and 2nd phase: Anaemia (dizziness, headache); Mild puerperal fever

*Smilax glabra *Wall. ex Roxb. Smilacaceae	ຢາຫົວ Ya Houa	ຢາຫົວ Ya Houa	Kool 550; Kool 774; Kool 782; Kool 824	Tuber	Roast-decoction-drink	Postpartum recovery 1st and 2nd phase: Anaemia (dizziness, headache); Mild puerperal fever

*Syzygium antisepticum *(Blume) Merr. & L.M.Perry Myrtaceae	ສະເມັກ Samek	ກວາດ ຄາເມັກ Kouad Khamek	VL 1376; VL 1768	Innerbark	Roast-decoction-drink	Postpartum recovery 1st and 2nd phase: Puerperal fever, Anaemia, Blood loss, Weakness

*Tacca chantrieri *André Taccaceae	ເຟັ້ຍຟານ Phiaphane	ຕຸຍ Toui	VL 1738; VL 1786; VL 1822; VL 1859; VL 2033; VL 2046; Kool 505	Whole plant	Decoction-drink	Postpartum recovery 1st phase: Postpartum secondary bleeding; Perineal healing, Retraction of the uterus; Expel lochia; Abdominal pain

*Tamarindus indicus *L. Fabaceae	ໝາກຂາມ Mak Kham	ຂາມ Kham	VL 2126	Stems; Leaves	Steambath; Decoction-wash	Postpartum recovery 2nd phase: Varicella, Mild puerperal fever; Neonatal rash after high fever

*Tetracera scandens *(L.) Merr. Dilleniaceae	ສ້ານດິນ Sandin	ກສິ ບໍລໍ Ksi Borlor	VL 2048; VL 1408	Roots	Roast-decoction-drink; Fresh- chew	Postpartum recovery 1st phase: Postpartum secondary haemorrhage, Aperative; Infant oral candida

*Trevesia palmata *(Lindl.) Vis. Araliaceae	ຕ້າງ Tang	ເກຼິລ ຕາງ Kreuale Tang	VL 2032; VL 1740; VL 1835	Stems; Roots	Roast-decoction-drink	Postpartum recovery 1st and 2nd phase: Perineal healing, Retraction of the uterus; Expel lochia; Abdominal pain; Physical recovery; Lactagogue

*Uncaria macrophylla *Wall. Rubiaceae	ຂໍເບັດ Kho Bet	ກສິຂໍເບັດ Ksi Kho Bet	VL 1795; VL 1875	Stems; Leaves	Decoction-drink	Postpartum recovery 1st phase: Postpartum apertive

*Zea mays *L. Poaceae	ສາລີ Sali	ສາລີ Sali	Cultivated	Corn	Roast-decoction-drink	Postpartum recovery 1st phase: Delivery abdominal pain; Expel lochia
*Zingiber officinale *Roscoe Zingiberaceae	ຂິງແດງ; ປີດິນ Kingdeng, Pidin	ໄຕ ຈເຣີ Tai Sheure	VL 1827	Leaves	Decoction-drink; Roast-poultice-message	Postpartum recovery 1st and 2nd phase: Anaemia (dizziness, headache), Mild puerperal fever; Lactagogue; Lactagogue, Painful or hard breasts, Improve flow of milk

*Ziziphus funiculosa *Ham. Rhamnaceae	ກຳລັງເສີອໂຄ່ງ Kamlang Seuakong	ກຳລັງເສີອໂຄ່ງ Kamlang Seuakhong	VL 1347; VL 1426; VL 1563; VL 2044; Kool 546	Bark; Roots	Roast-decoction-drink	Postpartum recovery 1st and 2nd phase: Anaemia (dizziness, headache), Mild puerperal fever; Physical recovery

*Ziziphus oenoplia *(L.) Mill. Rhamnaceae	ເລັບແມວ Lep Miou	ກສິ ເລັບແມວ Ksi Lep Miou	VL 1365; Kool 680	Stems	Roast-decoction-drink	Postpartum recovery 1st and 2nd phase: Expel lochia; Postpartum abdominal pain; Perineal healing, Retraction of the uterus; Physical recovery

**Table 2:** Kry postpartum decoctions prepared with mixtures of medicinal plants^a^

Species in mixture^b^	Expel lochia	Postpartum secondary haemorrhage	Postpartum recovery	Postpartum anaemia	Puerperal fever	Postpartum varicella	Postpartum headache	Perineal healing	Rectraction of the uterus	Lactagogue	Infant varicella	Infant is late walking
*Alpinia galanga* *Zingiber officinale*			1;2;3	1;2;3	1;2;3		1;2;3			1;2;3		
*Blumea balsamifera* *Cissus repens*												4
*Embelia ribes* *Castranopsis indica*		2;3	2;3					2;3	2;3			
*Lagerstroemia calyculata* *Choerospondias axillaris*	1;2;3	1;2;3	1;2;3	1;2;3				1;2;3	1;2;3			
*Trevesia palmata* *Psychotria sarmentosa*			1;2;3					1;2;3	1;2;3	1;2;3		
*Rubus cochinchinensis* *(alt. Rubus tonkinensis)* *Smilax glabra* *Ziziphus funiculosa*			2;3	2;3	2;3		2;3					
*Catunaregam spathulifolia* *Amaranthus spinosa*						2;3					4	

^a^Numbers in cells denote postpartum phase: 1. Delivery hut, first pp phase; 2. Menstruation hut, second pp phase; 3. Main house, third pp phase; 4. Infant healthcare. ^b^See Table 1 for full scientific names and Lao and Kry names

### Menstrual cycle

**Figure 3:**
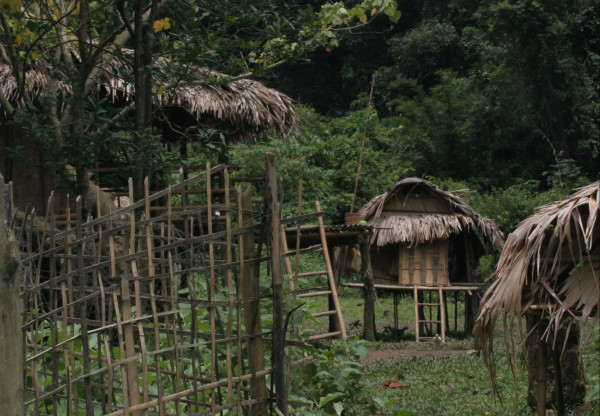
**Kry menstruation hut at Ban Maka Tai**. Note the woman sitting by the hut. Photograph by V. Lamxay (June 2008).

Spatial taboos are common among the Kry, and people may need to stay on the ground, are allowed only to enter their own house, or are required to stay in a special house away from the house or village. The menses invokes such a taboo and Kry women are obliged to stay in a little hut placed a short distance away from the main house (Figure 3). During the menses women stay, eat and sleep in this hut, and are prohibited from entering any houses in the village, including their own. Infants, until they are weaned (at 9 - 12 months), are taken with the mother to the menstruation hut. Normal labor, such as working in the rice fields, or household chores, like pounding rice, cooking, and washing are also prohibited. No people, including close relatives, are allowed to enter the hut, or touch the mother and infant, either in the hut or outside it, during this period. The mother is free to move about outside the hut in the village or go to the river for washing, but is not allowed to go to the forest to collect food, or leave to another village. At the end of her period the mother moves back to her house.

The Kry Phong observe the same spatial taboos, but the location of the menstruation is radically different. The menstruation huts forms an annex of the main house, which is reached from the ground by means of a ladder separate from the main ladder. The menstruation annex is used in a similar manner as the independent menstruation hut, and like the menstruation hut requires rebuilding every 5 year or so.

### Pregnancy

Pregnancies are a common aspect of life for women in reproductive age where having up to 12 pregnancies is not uncommon, and most families have 5 - 7 living children. Pregnancy is not strongly associated with spatial taboos, and the mother continues her normal work in the fields and around the house. At the end of the pregnancy if the mother becomes inconvenienced she will abandon her work, and stay at home in the village together with her husband, until the onset of labor. Around this time the husband will collect medicinal plants used during delivery and the first postpartum phase. Use of medicinal plants, either in steambaths or consumed in decoctions, is avoided during pregnancy. The only advice reported was dietary, and recommended daily consumption of banana plant pseudostems or young leaf-sheaths (*Musa acuminata *Colla), as it would guarantee an easy delivery.

### Delivery hut, delivery and first postpartum phase

**Figure 4:**
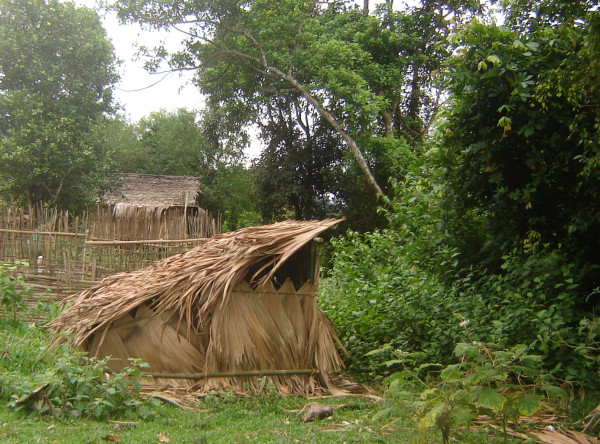
**Kry delivery hut at Ban Maka Tai**. The river lies beyond the shrubs to the right. Photograph by V. Lamxay (June 2009).

Towards the end of the pregnancy the husband will construct a makeshift hut by the river, downstream from the village and regular bathing places, for delivery (Figure 4). At the onset of labor, the mother and her husband will move to the makeshift hut, and remain there for delivery. Direct relatives take care of the couple's other children. The mother gives birth, and does not leave the hut until after a trial period of 5 days. The husband assists with the delivery, and other people may give advice, but are not allowed to enter the hut or touch the parturient mother. Following delivery, the umbilical cord is tied with a bamboo fiber string, which was peeled from the inside of the cane in long strips and twisted into a string, as it is deemed cleaner than regular cotton string (several species in the genus *Bambusa *can be used). The umbilical cord is then cut by either father or mother, using a freshly cut splinter of fresh bamboo cane of *Gigantochloa parvifolia *(Brandis ex Gamble) T.Q. Nguyen. The husband subsequently buries the placenta after expulsion in a shallow pit near the hut. Directly after delivery the mother will also start breast-feeding the infant. The husband has a key role in facilitating childbirth, and is the only person to touch the mother, support her, collect water, make fire, boil water, or supply food and medicinal plants. Water can be fetched from the river and can be used, either cold or heated, for washing the neonate. Medicinal plants are collected either by the husband in the vicinity of the hut, or supplied by relatives if collected from further away. The mother is not allowed to bathe or cleanse herself until the fifth day postpartum, but can change clothes and sheets. During the time that the husband assists his wife at the delivery hut, he may only enter his own house, as a taboo rests on entering other houses in the village.

To reduce abdominal pain directly following parturition the mother drinks about a quarter of a liter of water boiled with a spoonful of salt. During the whole first day she will also drink a hot decoction of lightly roasted corn (*Zea mays *L.) to reduce abdominal pain and aid expulsion of lochia. Starting the day following parturition the mother eats a special diet consisting of small amounts of rice cooked with salt. This diet is complemented with cooked banana inflorescences (*Musa acuminata *Colla) and rattan shoots (*Calamus rudentum *Lour.) as lactagogue. In addition the mother will drink a variety of hot decoctions to aid in postpartum recovery (Table 1 &2): *Tacca chantrieri *André to aid healing of the perineum, retraction of the uterus, expulsion of lochia, and reducing abdominal pain; a mixture of *Trevesia palmata *(Lindl.) Vis. and *Psychotria sarmentosa *Blume to aid healing of the perineum, retraction of the uterus, as a lactagogue, and for general postpartum recovery; a mixture of *Zingiber officinale *Roscoe and *Alpinia galanga *(L.) Willd. to protect and reduce postpartum fever or dizziness resulting from postpartum anaemia, and as a lactagogue; and a mixture of *Lagerstroemia calyculata *Kurz and *Choerospondias axillaris *(Roxb.) B.L.Burtt & A.W.Hill to aid healing of the perineum, retraction of the uterus, expulsion of lochia, reduce postpartum fever, reduce dizziness resulting from postpartum anaemia, and in case of postpartum secondary haemorrhage.

After five days the mother, father and infant cleanses themselves at the hut, and the mother moves with the infant to her menstruation hut near the house, after which the husband destroys the makeshift hut by the river.

Unlike many other cultures in Southeast Asia [12,26-33], the Kry do not practice the use of *hotbeds *or *mother roasting *during postpartum recovery. The use of *hotbeds *is practiced by the Kry Phong, and thus not considered to be traditional, but borrowed from the neighboring ethnic groups. Like the Kry, the Kry Phong use a specifically constructed delivery hut, but after delivery the mother lies on a bamboo bed covered with leaves of *Blumea balsamifera *(L.) DC., some 30 cm above the ground, heated from below using an open charcoal brazier.

### Complications during pregnancy and parturition

In case of miscarriage (intrauterine fetal death) leading to spontaneous expulsion of the fetus the mother goes to the river downstream of the village, and her husband constructs a makeshift delivery hut. She stays there for a period of 5-10 days, to expel the fetus and lochia, and remains until all postpartum bleeding has ceased. At this makeshift 'miscarriage' hut, she is tended to by her husband, which in this situation is not allowed to enter. The mother will drink a decoction of the roasted root and stem of *Rhapis laosensis *Becc. to stop miscarriage postpartum bleeding; and a decoction of the leaves of *Blumea balsamifera *(L.) DC. is consumed to accelerate physical recovery. After regaining health and cleansing the body she returns directly to the family's house, and resumes normal work.

Informants did not report having experienced fetus malpresentations (i.e. breech birth), nor complications involving failure of placental expulsion or general complicated deliveries. Informants did report that if the delivery was protracted that experienced mothers were called in for advice, followed by the local trained midwife from a village 4 hours downstream. The local trained midwife has very basic equipment, such as a stethoscope, but lacks medical supplies such as synthetic oxytocin, antibiotics, sterile suturing material that may aid during a critical delivery. Transport of the parturient mother to the nearest medical post would take several days.

### Second postpartum phase

Instead of *hotbed *or *mother roasting*, the mother and infant move from the delivery hut by the river to the menstruation hut close to the house for a second phase of postpartum recovery. This phase lasts anything from 8 - 15 (or 30) days depending on the recovery, and is determined by the termination of postpartum bleeding. Once arrived at the hut visiting is no longer restricted to only the closest kin.

At the menstruation hut the mother helps herself to cook and boil water, and takes care of her infant. The husband assists by collecting medicinal plants and supplying food and water, but may not enter the hut. The mother cleanses herself as often as she prefers, but at least once a day, and washes the infant 1 - 3 times/day.

As during the first postpartum phase, a special diet is prescribed consisting of small amounts of rice cooked with salt, complemented with cooked banana inflorescences (*Musa acuminata *Colla) and rattan shoots (*Calamus rudentum *Lour.), as a lactagogue. Fish can also be eaten, with the exception of catfish. In addition to the postpartum hot decoctions discussed above, the mother also drinks the following hot decoctions: a mixture of *Castanopsis indica *(Lindl.) A.DC. and *Embelia ribes *Burm.f. to reduce abdominal pain, expel lochia, postpartum secondary haemorrhage, perineum healing, and retraction of the uterus; a mixture of *Rubus cochinchinensis *Tratt. (alternatively *Rubus tonkinensis *F.Bolle), *Smilax glabra *Roxb. and *Ziziphus attapoensis *Pierre to aid postpartum recovery, alleviate postpartum anaemia and headaches, and treat puerperal fever and postpartum varicella; a mixture of *Catunaregam spathulifolia *Tirv. and *Amaranthus spinosus *L. in case of postpartum varicella of mother or infant (cf. Table 1 &2).

The Kry Phong second phase of postpartum recovery is practiced analogous to that of the Kry, except that the mother and infant reside in the menstruation annex of the main house.

### Third postpartum phase

The mother and infant move to the main house when all postpartum bleeding has ceased, and the mother and infant have been resolved of their blood contamination in a spiritual ceremony. At this time, the father also formally gives the infant a name. The mother can then resume her normal chores, including the care of the older offspring.

A spatial taboo in the third postpartum phase prohibits the mother and infant from entering into the parental bedroom for a period of at least 1 month, or until postpartum bleeding has fully ceased. The mother and infant sleep in the main room, close to the fire to warm the infant, until they are allowed to move to the parental bedroom. Sexual intercourse is avoided for a period of 3-4 months.

The same diet is eaten as during the second postpartum phase, with a continued focus on plant species that reportedly enhance breast-milk supply. Meat, such as chicken, pork, dogs and beef are avoided, and so is cat-fish. The mother continues to consume the second phase postpartum hot decoctions, discussed above, in the third phase, depending on her needs (cf. Table 1 &2).

### Cultural consensus

**Figure 5:**
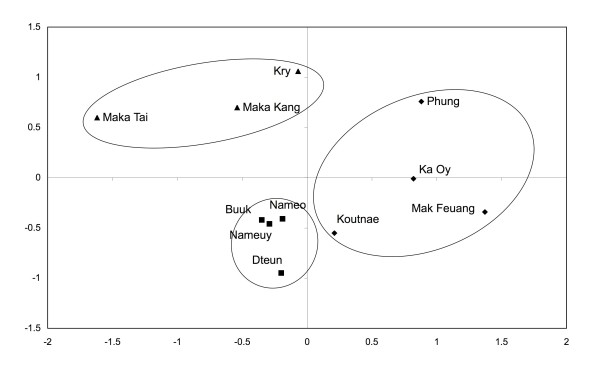
**Similarity in postpartum plant use in the Nam Noi and Nam Pheo watersheds**. Similarity is plotted using non-metric multidimensional scaling (Kruskal Stress 0.111). Data combines the in-depth study of Kry childbirth plant use with Brou, Saek and Kry postpartum data [15]. Distances between points are relative similarities. Points represent individual village datasets, and for 'Kry' the overall postpartum-used plants from this study. Icons represent: Brou (Diamond), Saek (Square), and Kry (Triangle). Clustering of ethnic groups is evident.

De Boer and Lamxay [15] reported on a comparative study of medicinal plant use in women's health of the Brou, Saek and Kry; and analyzed the cultural consensus between the three ethnic groups and rejected the hypothesis that these groups belong to a single culture of plant use. Plotting of the different villages and ethnic groups using multi-dimensional scaling resulted in a clear independent clustering of each ethnic group. Re-analysis of that data, using the current Kry data as an additional dataset parallel to previous data, corroborated the findings of the previous study (Figure 5).

## Discussion

### Pregnancy and childbirth

Pregnancy and childbirth in most societies is perceived as a period of vulnerability [11]. In Laos this view is generally shared, but many ethnic groups also see pregnancy and childbirth as a normal event and not a cause of concern [34]. The Kry side with the latter, and believe that a continuation of the normal daily routine by the mother facilitates delivery.

Dietary restrictions during pregnancy are reported from other ethnic groups in Laos: the Hmong, Phunoi, Phutai and Khmu avoid eating too much for fear of having a big baby [34,13]; the Khmu avoid taro and sweet potatoes as it would make the mother fatter, which complicate delivery [34]; the Akha mention that wild animals, fermented foods, and pumpkin leaves are avoided during pregnancy [13]; and the Hmong and Khmu avoid meat killed by tigers [13]. The Kry do not have a special diet during pregnancy, but medicinal plants, modern medication, and alcohol are avoided. Whether these restrictions are based on traditional perceptions of teratogenicity or abortive properties, or influenced by external biomedical knowledge from health workers, would be interesting to study.

Kry mothers in labor are aided by their husbands, with advice from a traditional birth attendant, typically an older woman with multiple children. Any mother having survived multiple childbirths attains the informal role of birth attendant, and has a key part in vertical transmission of knowledge on childbirth. She will tell the couple which plants to collect, how to prepare them, and how often to consume them. In addition she can sooth anxiety by delving from her experience. Kry childbirth takes place in a special delivery hut, away from the main house, and generally from the village too. The Brou and Katang have also been reported to practice this type of ex-situ homebirth, but these customs are quickly loosing prevalence [35]. The Brou are reported to have childbirth take place in special birthing huts, placed near the house, where the mother will stay up to 3 days after giving birth [19]. Katang women in labor traditionally leave the village and give birth in the forest supported by their husband and a traditional midwife. A small camp with a fire is set up for use during parturition and the first few days postpartum, however during the rainy season a makeshift hut may be constructed for shelter [35]. The practice of giving birth outside the village, and remaining there for a period of 3 - 5 days can be viewed as a viability test of mother and infant, and in the case of the Kry the child is not given a name until they have moved to the main house.

The Kry will breast-feed the infant immediately after birth, which is in contrast with many other ethnic groups in Laos that discard the colostrum and postpone the first breast-feeding [12,13].

### Postpartum period

The postpartum period is important in many Southeast Asian cultures, and is seen as a period of recovery and often entails a period of confinement ranging from 10 up to 45 days. In accordance with humoral medicine pregnancy is seen as a hot state; with parturition heat is lost and the woman comes into a state of excess cold, and during the postpartum period care should be taken to restore the mother to equilibrium [36]. Confinement as a treatment involves staying inside and near heat, washing only with hot water, drinking hot drinks, eating hot food, and staying away from draughts [36]. Confinement as a term is fairly broad and can include steam bath and bathing, *mother roasting*, dietary prescriptions and proscriptions, and consumption of medicinal plant decoctions.

Ritual behaviors are important in everyday Kry life [20], and confinement is an integral part of those rituals. Confinement and postpartum recovery are both considered important and strictly observed, and failure to do so would have far-reaching consequences. The spatial restrictions of confinement include the delivery hut (first phase), the menstruation hut (second phase) and sleeping outside the parental bedroom in the main house (third phase).

### Steam bath and cleansing

Postpartum steam bath is common throughout Southeast Asia (for review see [36]). Postpartum cleansing is important to the Kry, but the use of steambaths or washing with hot decoctions is not practiced. The Kry further deviate from humoral medicine in that the mother can cleanse herself using cold water. In addition, the mother cleanses herself first five days after childbirth, before moving from the delivery hut to the menstruation hut. This seemingly unsanitary practice might have its base in the past when uncontaminated boiled water was inaccessible, and cleansing could lead to infections.

### Diet, proscriptions and prescriptions

Diet is important during pregnancy, confinement and in some cultures also after confinement [8]. A 2007 survey in Vientiane, Lao PDR, found that 93% of respondents observed a strict diet after delivery [12]. Postpartum avoidance of foods classified as cold, such as fresh fruits and vegetables, cold foods, and plain water, is almost universal. Many ethnic groups report the prescription of hot decoctions of ginger (*Zingiber officinale *Roscoe) or turmeric (*Alpinia galanga *(L.) Willd.), boiled rice, boiled vegetables and boiled chicken or fish, all combined with salt for drying out the womb [36].

The border between food and medicine can be vague when studying plant use of people that source the majority of their vegetables (e.g. fruit, flowers, nuts, herbs, shoots, tubers, fronds, leaves, pods) and medicines (e.g. roots, bark, leaves, herbs, shoots) from the wild. Kry respondents considered the consumption of a hot decoction of roasted corn medicinal, but the consumption of specific rattan shoots and wild banana inflorescences to stimulate breast milk production as food. Dietary prescription is explicit in the first postpartum phase in the delivery hut, and the mother eats mainly rice cooked with plenty of salt, as well as some vegetables to induce and stimulate breast milk production. Starting from the second postpartum phase dietary prescriptions ease and other vegetables can be eaten, as well as most fish. However the staple remains rice with salt.

### Medicinal plant use

Medicinal plant use is an integral part of cultural traditions during postpartum recovery. The 49 species reported in this study are likely to be a close approximation of current postpartum plant use as information was gathered through multiple interviews over multiple years using both female and male informants and interviewers.

Most of the medicinal plants in this study can be found in scientific literature reported for the same or similar affections. The hypotheses whether or not these traditional remedies have effective medicinal properties; and whether the reported taxa have comparable medicinal properties at genus, species or genotype level, lie beyond the scope of this research. De Boer & Lamxay [15] report the postpartum use of 29 of these species (59% of species), and all species reported for the Kry in that study were confirmed here. Perry's Medicinal Plants of East and Southeast Asia [37], an exhaustive review of scientific literature on medicinal plant use up to 1961, reports the traditional use of 65% of the species, with 53% used in similar ways in women's healthcare. At genus level the documented uses share even higher similarity, 100% for traditional use, and 88% for women's healthcare. The species not reported in Perry [37], or closely-related taxa, can found in other literature and are briefly discussed below.

The postpartum use of *Ageratum conyzoides *L. is reported by Manderson [26] in Peninsular Malaysia, and by Liulan et al. [1] for the Haw ethnic group in Northern Thailand. *Amomum *spp. are commonly used in postpartum healthcare in Southeast Asia [37], but the use of *Amomum microcarpum *C.F.Liang & D.Fang is only reported by de Boer & Lamxay [15]. Two taxa closely related to *Barringtonia longipes *Gagnep. are reported in women's health: *B. acutangula *(L.) Gaertn. is reported in treatment of blenorrhea and menorrhagia from Cambodia [38], and as a contraceptive and abortificient from Peninsular Malaysia [39]; and *B. edulis *Seem. in parturition in the North Solomons [40] and as a contraceptive in Vanuatu [3]. Four taxa closely related to *Callicarpa arborea *Roxb. are reported in women's health: *C. candicans *(Burm.f.) Hochr. in postpartum recovery to stimulate the appetite from Indo-China [41], as an emmenagogue in Indonesia [42]; and to treat abdominal pains in Peninsular Malaysia [43], an application very close to that reported here; *C. longifolia *Lam. as used in a postpartum decoction [43]; *C. pedunculata *R.Br. as an emmenagogue and postpartum depurative in Indonesia [42]; and *C. bodinieri *Levl. as an emmenagogue and to treat blenorrhea in China [44]. *Castanopsis indica *(Lindl.) A.DC. is reported in Perry, but *C. sclerophylla *Schottky is used to arrest haemorrhage in puerperal women [45]. A hot infusion of the bark of the *Catunaregam spinosa *(Thunb.) Poir., a taxon closely related *C. spathulifolia *Tirv., is used to regulate the menses in Indo-China [41]. The monotypic *Choerospondias axillaris *(Roxb.) B.L.Burtt & A.W.Hill is reported as a small-scale NTFP in Nepal for its edible fruit [46], and used in Vietnam to treat burns [47]. *Cissus repens *Lam. is not recorded in postpartum use, but used traditionally to allay headaches [39,48]. A closely related taxon, *Cissus repanda *Vahl, is used during postpartum recovery by the Yao and Hmong ethnic groups in Northern Thailand [49]. Two taxa from Southeast Asia related to *Diospyros apiculata *Hiern are used in women's healthcare: *D. decandra *Lour. is used as emmenagogue in Cambodia [38]; and *D. malabarica *(Desr.) Kostel. to treat vaginal discharge in Indo-China [41]. *Embelia ribes *Burm.f. is reported as a taeniafuge in Indo-China [41], and the leaves are reportedly toxic and applied topically to treat pimples and skin eruptions [50]. *Glochidion eriocarpum *Champ. is used medicinally without further specification in China [37], but *G. macrocalyx *J.J.Sm. is given to women in parturition in Indonesia [42]; and *G. cauliflorum *Merr. is given to women to promote delivery in the Philippines [37]. *Gonocaryum lobbianum *(Kurz) Miers is used by the Karen in Northern Thailand to alleviate pain and during postpartum [49], and by the Yao in Northern Thailand for abdominal pain and for treating obstetric diseases [51]. *Lagerstroemia calyculata *Kurz is not mentioned by Perry [37], but is reported as part of a postpartum remedy of the Lahu in Northern Thailand [52]. *Mallotus paniculatus *(Lam.) Muell.-Arg. a closely related taxon to *M. barbatus *Muell.-Arg. from Southeast Asia, is used as postpartum protective in Peninsular Malaysia [39]. *Neonauclea sessilifolia *(Roxb.) Merr., a taxon related to N. purpurea (Roxb.) Merr., is used for postpartum recovery in Cambodia [38]. *Phoebe lanceolata *(Nees) Nees, nor any of its closely related taxa, are reported by Perry [37] for postpartum use. However its use as a vulnerary is documented from India [53], and its use in postpartum medicine by the Brou and Saek ethnic groups in Laos [15]. *Polyalthia cerasoides *Benth. & Hook. is used by the Lahu in Northern Thailand to treat hemorrhoids [52], and *P. hypoleuca *Hook.f. & Thomson is used as a postpartum protective in Peninsular Malaysia [39,43]. *Rhapis excelsa *(Thunb.) Henry is the only species in the genus with reported medicinal use, and is used as hemostatic, antidysenteric, and circulatory [37]. *Syzygium antisepticum *(Blume) Merr. & L.M.Perry (syn. *Syzygium gratum *(Wight) S.N.Mitra) has not been previously reported in postpartum recovery, but other taxa in *Syzygium *from Southeast Asia have: *S. aromaticum *(L.) Merr. & L.M.Perry [2,39,54]; *S. cumini *(L.) Skeels [52,55]; *S. kinabaluensis *(Stapf) Merr. & L.M.Perry [56]; *S. leptostemon *(Korth.) Merr. & L.M.Perry [43]; *S. longiflorum *Presl [39]; and *S. polyanthum *(Wight) Walp. [2]. *Tacca palmata *Blume, closely related with *T. chantrieri *Andre, is used in the Philippines as a remedy for menstrual disorders [57]. *Trevesia palmata *(Lindl.) Vis. is reportedly used in Peninsular Malaysia in the treatment of fractured bones and skin complaints [39,43]. Use of *Uncaria macrophylla *Wall. in traditional medicine is not reported, but *U. sessilifructus *Roxb. is used by the Yao in Yunnan to treat abdominal pain and hysteritis [58]. Three taxa from Southeast Asia related to *Ziziphus funiculosa *Ham. are reported in women's healthcare: *Z. rugosa *to treat menorrhagia in Burma [59]; *Z. oenophila *Mill. in neonatal feber in Vietnam [41]; and *Z. kunstleri *King as a postpartum protective medicine in Malaysia [39,43].

## Conclusions

Plant use is common during postpartum recovery among the Kry ethnic group. Observing a period of confinement for the mother and newborn infant is common during which a variety of treatments are practiced, such as drinking herbal decoctions and infusions, and abiding by food proscriptions and prescriptions. These treatments and the plant species used in the treatments aim to relieve postpartum abdominal pain, reduce postpartum haemorrhage, aid in physical recovery, augment lactation, and treat illness in infants. Knowledge of the plant species used, where to collect them, how to harvest them, how to prepare them and how to use them is an important realm of knowledge possessed by women in these communities, but shared with men.

Modernization of healthcare in Laos could benefit from integrating aspects of traditional practices and plant use into healthcare modernization programmes through active involvement of local people. It would facilitate the implementation of culturally appropriate healthcare that respects traditional knowledge and contributes to bio-culturally sustainable development.

In addition, there is a need for ethnobotanical research into the rich biocultural diversity of the ethnic groups in Southeast Asia as rapid assimilation with mainstream culture increases. Research focusing on traditionally ignored subjects such as women's health care are scarce [1,2,15], and general ethnobotanical studies often overlook the variety and relative importance of plants used in women's healthcare [60-64], with a few notable exceptions [65-68,49,51,54,58].

Research focusing on the pharmacological mechanisms and the efficacy of these treatments, that are both ancient and widespread, could provide insights that could help to augment and improve both local and Western postpartum care.

## Competing interests

The authors declare that they have no competing interests.

## Authors' contributions

VL, HdB and LB conceived the research. VL was responsible for field research and interviews. VL and HdB identified the herbarium vouchers; and processed the data. HdB performed the quantitative analysis. VL and HdB drafted the manuscript. All authors have read and approved the final manuscript.
